# Removal of Contaminant DNA by Combined UV-EMA Treatment Allows Low Copy Number Detection of Clinically Relevant Bacteria Using Pan-Bacterial Real-Time PCR

**DOI:** 10.1371/journal.pone.0132954

**Published:** 2015-07-14

**Authors:** Bruce Humphrey, Neil McLeod, Carrie Turner, J. Mark Sutton, Paul M. Dark, Geoffrey Warhurst

**Affiliations:** 1 Institute of Inflammation and Repair, Faculty of Medical and Human Sciences, University of Manchester, Manchester, United Kingdom; 2 Infection, Injury & inflammation Research Group, Salford Royal NHS Foundation Trust, Salford, United Kingdom; 3 Public Health England, Microbiology Services Division, Porton Down, Salisbury, United Kingdom; University of Illinois at Chicago, UNITED STATES

## Abstract

**Background:**

More than two decades after its discovery, contaminant microbial DNA in PCR reagents continues to impact the sensitivity and integrity of broad-range PCR diagnostic techniques. This is particularly relevant to their use in the setting of human sepsis, where a successful diagnostic on blood samples needs to combine universal bacterial detection with sensitivity to 1-2 genome copies, because low levels of a broad range of bacteria are implicated.

**Results:**

We investigated the efficacy of ethidium monoazide (EMA) and propidium monoazide (PMA) treatment as emerging methods for the decontamination of PCR reagents. Both treatments were able to inactivate contaminating microbial DNA but only at concentrations that considerably affected assay sensitivity. Increasing amplicon length improved EMA/PMA decontamination efficiency but at the cost of assay sensitivity. The same was true for UV exposure as an alternative decontamination strategy, likely due to damage sustained by oligonucleotide primers which were a significant source of contamination. However, a simple combination strategy with UV-treated PCR reagents paired with EMA-treated primers produced an assay capable of two genome copy detection and a <5% contamination rate. This decontamination strategy could have important utility in developing improved pan-bacterial assays for rapid diagnosis of low pathogen burden conditions such as in the blood of patients with suspected blood stream infection.

## Introduction

Molecular diagnostics aimed at the rapid detection of infectious diseases have become a powerful tool in modern medicine, with over 160 products currently approved by the FDA. An overwhelming majority of these tests are for clinical syndromes with very narrow aetiological spectrums, targeting single organisms or small, restricted panels of pathogens [1]. Diagnosis of diseases such as human sepsis, where the causative agents in circulating blood display significant genetic diversity, presents a much greater challenge. A pan-bacterial approach, such as detection of conserved ribosomal RNA sequences, must be employed if infection is to be confirmed or excluded with certitude, but broad-range primers are prone to detecting contaminant microbial DNA invariably present in the PCR reagents themselves. In some cases, such contamination cannot easily be discriminated from invading organisms based on quantitative thresholding. For example, the blood of patients with sepsis may have a low pathogen burden (~1–3 CFU/ml) [2]. Methods using species-specific probes can partially circumvent this issue and also provide some information to guide early stage antimicrobial therapy, but cannot detect or exclude infections caused by organisms not present on their restricted panels. Furthermore, taxonomic identification by itself is of increasingly limited use without an accompanying antibiotic susceptibility profile. Reagent contamination is therefore a major barrier to the production of an effective PCR diagnostic for sepsis.

Broad-range PCR techniques have long been known to generate products in negative control reactions to which no template DNA has been added [3]. Common sources of contamination are environmental, or the result of product carryover from previous reactions performed targeting the same amplicon, but these can be reduced effectively through prudent laboratory management and surface decontamination [4]. Much more problematic is the inherent presence of bacterial DNA in PCR reagents themselves. With its high affinity for DNA, Taq polymerase is particularly prone to contamination, not only with DNA from its recombinant production host (commonly *E*. *coli*), but also any traces of DNA from environmental organisms in the water and buffers used during its purification [3,5–7]. Other PCR components, including commercially obtained primers, water, and plasticware have all been observed as sources of contamination [8,9]. Even ‘DNA-free’ reagents often come with a caveat limiting the quality assurance guarantee to a certain range of organisms, notably the recombinant host for polymerase expression, or a threshold level still above that expected in some clinical samples.

Researchers have employed various decontamination measures to overcome these problems, including enzymatic degradation, UV light, 8-methoxypsoralen, and filtration [7,10–14]. None have proved definitive, as the methods either suffer poor reproducibility or negatively impact PCR sensitivity [15,16]. A comprehensive multi-step procedure has been developed, but requires access to γ-radiation apparatus, which may be difficult for some to acquire [16]. One more recent alternative is the treatment of PCR reagents with photoreactive compounds ethidium monoazide (EMA) or propidium monoazide (PMA) prior to template addition [17–20]. The molecules intercalate any contaminating double-stranded DNA and form covalent bonds upon exposure to long wavelength light, preventing strand separation and subsequent PCR amplification. Whilst results presented with this method look promising, the absolute number of negative controls tested has not always been reported and inhibitory effects have been studied at template levels greater than the single figure copy numbers likely to be required for patient blood samples in the setting of sepsis. There exists, also, some disparity regarding the length of amplicon which can potentially be used with this technique, ranging from <200bp to >1kb.

The aim of the present study, therefore, was twofold; firstly, to determine the performance of previously described EMA/PMA based decontamination protocols in detection of low copy number bacterial DNA and secondly, to evaluate a new approach based on pan-bacterial real-time PCR with a simple reagent decontamination protocol combining UV and EMA treatment.

## Materials and Methods

### Genomic DNA isolation and dilution

Purified DNA from *E*. *coli* strain ATCC 11303 (Affymetrix) was used for assay optimisation work. Aliquots of a 100 ng/μl stock were diluted fresh prior to each experiment, in PCR grade water (Roche, Cat No. 03315932001) to minimise the chances of degradation at the femtogram levels used. Genomic DNA of the following strains was isolated using the Qiagen DNA Mini Kit (Qiagen, UK), according to manufacturer’s instructions: *Acinetobacter baumannii* (AYE), *Enterobacter aerogenes* (NCTC 10006), *Enterococcus faecalis* (NCTC 12697), *Haemophilus influenzae* (NCTC 12699), *Klebsiella pneumoniae* (MGH 78578), *Morganella morganni* (NCIMB 232), *Pseudomonas aeruginosa* (PA01), *Staphylococcus aureus* (NCTC 12493), *Staphylococcus epidermidis* (ATCC 35984), *Streptococcus pneumoniae* (NCTC 7465). DNA extractions were quantified using a NanoDrop Lite spectrophotometer (Thermo Scientific, UK), and diluted to desired concentrations prior to each experiment.

### Primers

Oligonucleotide primers were synthesised and HPLC purified by Sigma-Aldrich (Poole, UK). Upon arrival, lyophilised primers were resuspended in PCR grade water (Roche) to a master stock concentration of 100μM, and diluted further to 10μM working stocks. Oligonucleotide sequences are given in Table 1.

**Table 1:** Sequences of Primers Used in this Study.

Name	Position	Sequence (5’-3’)	Anneal	Extension	Reference
16S-F	942–963	TGGAGCATGTGGTTTAATTCGA	60°C	30s	[19]
16S-R	1082–1110	TGCGGGACTTAACCCAACA	60°C	30s	[19]
9F	9–27	GAGTTTGATCCTGGCTCAG	57°C	75s	[20]
1116R	1100–1116	YAAGGGTTGCGCTCGTT	57°C	75s	[20]
SF3c	347–364	GAGGCAGCAGTRGGGAAT	60°C	60s	This paper
SR5	1084–1102	GTTGCGGGACTTAACCCAA	60°C	60s	This paper

Nucleotide positions correspond to *E*. *coli* sequence (Genbank accession number J01859). Annealing temperatures and extension times are given for each primer pair.

### UV light exposure

Reagents were mixed as specified in 0.2 mL thin-wall PCR tubes (Appleton Woods, Cat no. BT101), and laid flat on the shelf of a Hoefer UVC5000 crosslinker. Exposure times ranged from 60–150 seconds, at a wavelength of 254 nm. PCR reactions with treated reagents were then set up immediately.

### EMA/PMA treatment

EMA (Biotium) was dissolved in ethanol to a concentration of 5mM under light controlled conditions, and aliquots stored at -20°C in amber tubes. Separate aliquots were also stored of PMA (Biotium), which was supplied as an aqueous 20mM stock, to minimise light exposure. For each experiment, a fresh aliquot was diluted in PCR grade water (Roche) to give a 50μM stock solution for further dilution in reagents to concentrations specified, in 0.5 mL Multiply tubes (Sarstedt, UK). Upon addition, the treated reagents were incubated in the dark on a metal cold block at 4°C for 10 minutes, and then exposed to 465–475 nm light in a PMA-Lite LED Photolysis Device (Biotium) for another 10 minutes, at room temperature. PCR reactions with treated reagents were then set up immediately.

### Real time qPCR

Prior to and following reaction set up, a PCR Workstation (Labcaire) was cleaned with a nucleic acid-degrading disinfectant (#TM306, Tristel, UK) and subjected to a half hour UV exposure. Tubes with PCR master mix, water and primers (where stated) sufficient for 8–10 x 25 μl reactions (total volume 200–250 μl) were prepared using Biosphere pipette tips (Sarstedt) for decontamination. Each 25 μl reaction comprised 12.5 μL AmpliTaq Gold 360 Master Mix (Applied Biosystems), 500nM each primer, 1.25 μl EvaGreen 20X fluorescent dye (Biotium) and PCR water (Roche) to a volume of 20 μl, with 5 μl PCR template. DNA template preparation and addition to reaction mix in 96 well PCR microplates (Axygen, Cat No. PCR-96-LC480-W) was performed in an SC-R Class II Microbiological Safety Cabinet (Labcaire). Real time monitoring of PCR was performed on a Roche Lightcycler 480 Instrument. For the SF3c-SR5 primer set, an initial 10 min denaturation at 95°C was followed by 40 cycles of: melting at 95°C for 10s; annealing at 60°C for 20s; extension at 72°C for 60s. Parameter alterations for other primer sets are shown in Table 1. PCR cycling was followed by melt curve analysis, with 3 acquisitions per °C while ramping from 55°C to 95°C at 0.19°C/s.

### Sequencing of contaminants

PCR reactions were purified using Microcon DNA Fast Flow PCR Grade Filters (Merck Millipore, Feltham, UK) to reduce the chance of post-reaction contamination, and diluted in PCR grade water (Roche). Sequencing was performed by Eurofins MWG Operon (Ebersberg, Germany) on an ABI3730XL dideoxy chain termination sequencing machine, using primers SF3c and SR5.

### Data access

The amplicon sequence data from this study have been submitted to GenBank under accession numbers KR611605—KR611615.

## Results

### Effect of amplicon length on qPCR reagent decontamination with ethidium monoazide

Efficiency of contaminant DNA inactivation by treatment with increasing concentrations of EMA was investigated for bacterial 16S ribosomal RNA (rRNA) gene amplicons of different sizes using two primer sets (16SF-R = 169bp [19] and 9F-1116R = 1108bp [20]) from two papers conducting similar work, and a newly designed pan-bacterial primer pair (SF3c-SR5) with an amplicon size of 756 bp (this study).

In the absence of EMA decontamination, 100% of no template control (NTC) reactions (n = 24) with the 169 bp and 756 bp amplicon primers generated bacterial products, as verified by melt curve analysis and agarose gel electrophoresis (Table 2). Nucleic acid sequencing of the amplicons often produced reads comprised of multiple peaks, suggesting that multiple contaminant species were present. Where clear single reads could be obtained, the products originated from a range of environmental species, indicative of reagent contamination, such as *Bradyrhizobium* spp. and *Sphingomonas* spp. At the other extreme, the 1108 bp amplicon generated product in only 4% of NTC reactions, but this was accompanied by reduced analytical sensitivity with only 75% and 13% detection of reactions spiked with 20 or 2 *E*. *coli* genome copies respectively (Table 2).

**Table 2:** Comparison of EMA Decontamination of Different Primer Sets.

	16SF-R (169 bp)			SF3c-SR5 (756 bp)			9F-1116R (1108 bp)		
[EMA]	NTC	20c	2c	NTC	20c	2c	NTC	20c	2c
**0** μ**M**	100%	100%	100%	100%	100%	100%	4%	75%	13%
**1** μ**M**	58%	100%	100%	4%	100%	75%	0%	25%	0%
**2** μ**M**	8%	100%	63%	0%	100%	50%	0%	13%	0%

Three primer sets of increasing amplicon length were treated with EMA and assessed for positivity in no template controls and detection of low level spiked *E*. *coli* gDNA. 24 no template controls (NTC) and 8 positive reactions of 20 genome copies (20c) and 2 genome copies (2c) each were performed for each EMA concentration per primer set.

Further studies examined the effect of EMA treatment of reaction components on the performance of the three primer sets. All reaction components were treated with EMA except for the EvaGreen dye, which was verified as free of bacterial contamination by separate testing, on the basis that it could interfere with light absorption. Following 2 μM EMA treatment, the smallest amplicon yielded 8% positives in NTC reactions, and 63% detection at the 2 genome copy level. The intermediately sized amplicon of 756 bp was more responsive to EMA decontamination, showing only 4% NTC contamination at 1μM EMA while retaining 75% detection of 2 spiked genome copies (Table 2). EMA treatment markedly inhibited the analytical sensitivity of the large 1108 bp amplicon with no detection of 2 *E*. *coli* genome copies following treatment with 1 μM or 2 μM EMA.

### Comparison of EMA and PMA decontamination of the SF3c-SR5 primer set

Further experiments were performed with primer set SF3c-SR5 to compare the effects of treatment with either EMA or PMA on contamination rates and low copy template detection. Again, reaction mixes were treated with varying concentrations of chemical, before addition of fluorescent dye and template.

**Fig 1:**
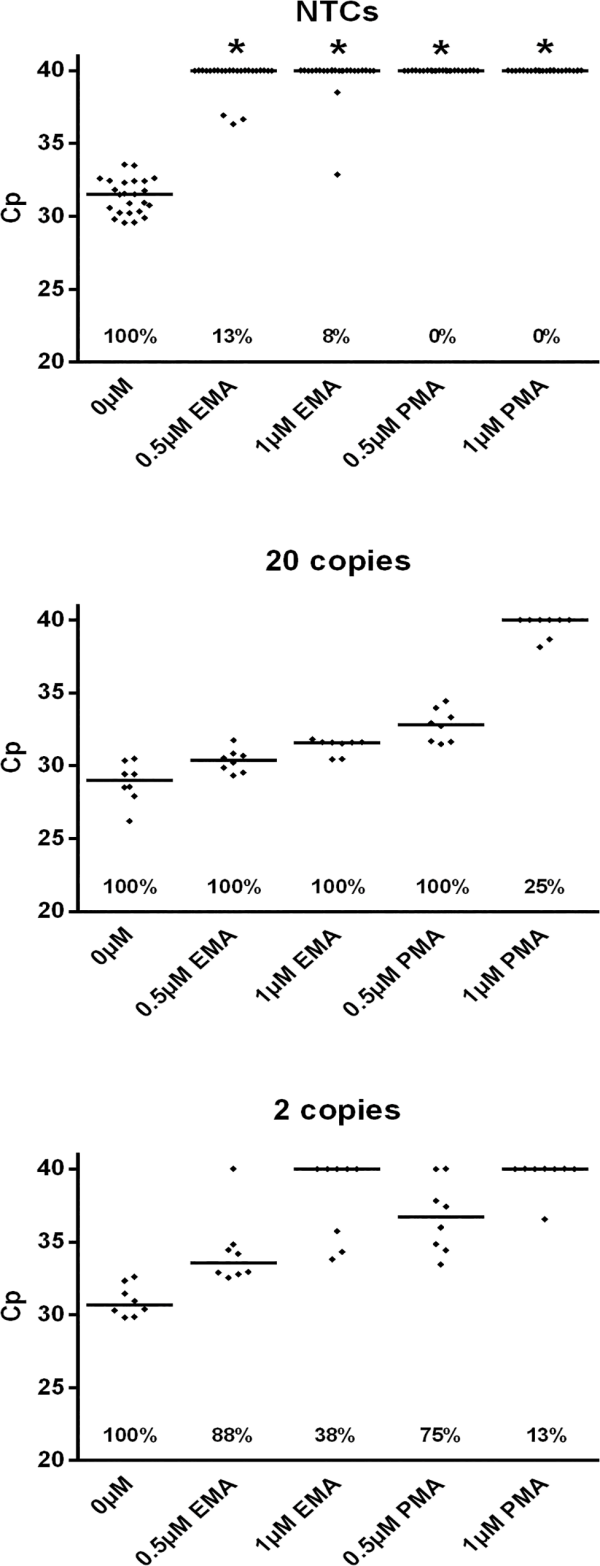
EMA and PMA Decontamination of SF3c-SR5 Primer Set. Master mixes of all reaction components (excluding EvaGreen dye) were treated with EMA or PMA before the addition of dye and template (PCR water in no template controls, and 20 or 2 *E*. *coli* genome copies for positives). 24 no template control reactions and 16 positive reactions (8 per template amount) were performed for each condition. Reactions that did not amplify within the 40 cycle threshold are represented as 40 for visual comparison. Effects of treatments on the number of positive NTC reactions, as compared to no treatment controls, were analysed statistically by Fisher’s exact test. * = P <0.0001. Horizontal bars = median value; percentages = number of positive reactions.

At the concentrations tested, PMA was the more potent chemical for decontamination of reagents, with 0% positive NTC reactions (n = 24) at both 0.5μM and 1μM, compared to 13% and 8% for EMA. However, PMA also had more of an inhibitory effect on detection of low copy templates, with later comparable crossing point (Cp) values, and fewer positive reactions when spiked with 2 *E*. *coli* genome copies (Fig 1). Based on these data, we concluded that treatment with either EMA or PMA alone was not sufficient to produce an assay with low contamination rates and high analytical sensitivity.

### Ultraviolet light exposure as a decontamination technique

Exposure of PCR reagents to ultraviolet light was investigated as an alternative decontamination technique. Fluorescent dye was again omitted during treatment, for fear of damage or interference with UV absorption.

**Fig 2:**
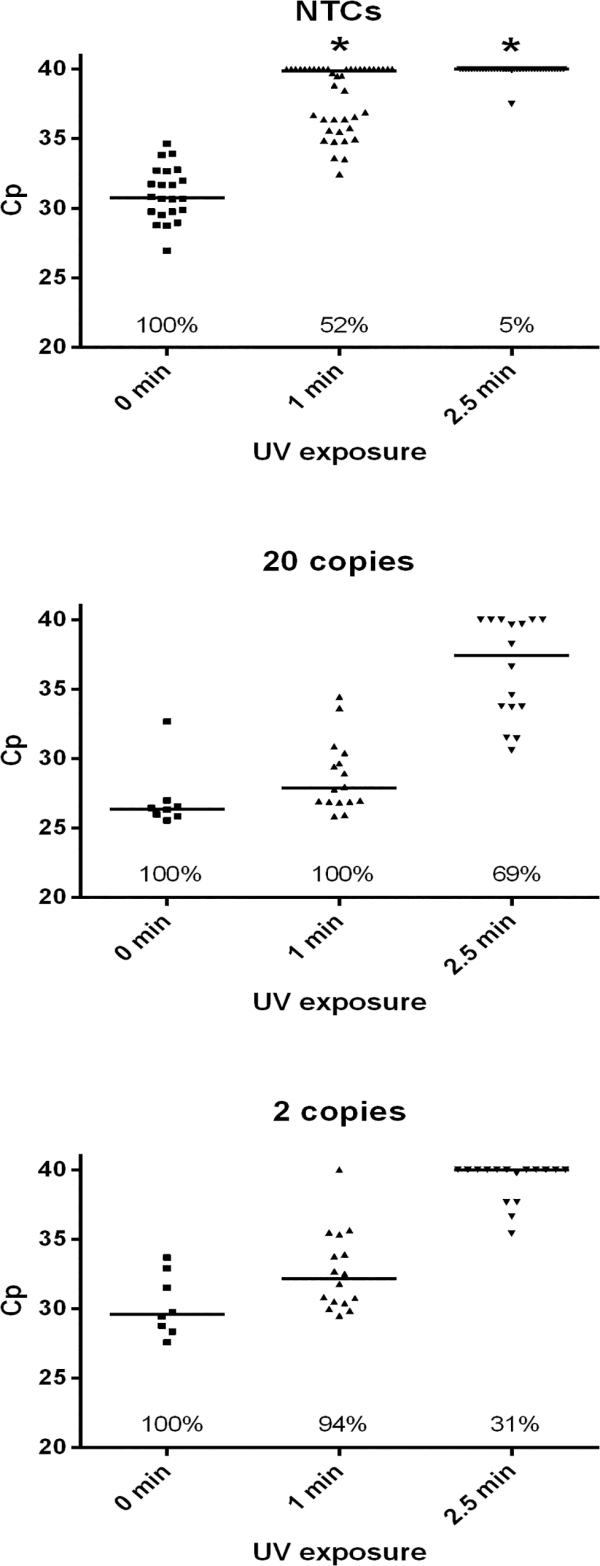
UV Decontamination of SF3c-SR5 Primer Set. Master mixes of all reaction components (excluding EvaGreen dye) were exposed to UV irradiation before the addition of dye and template (PCR water in no template controls, and 20 or 2 *E*. *coli* genome copies for positives). 42 no template control reactions and 32 positive reactions (16 per template amount) were performed for each UV condition, with 22 NTC and 16 positive reactions respectively for non-treated controls. Reactions that did not amplify within the 40 cycle threshold are represented as 40 for visual comparison. Effects of treatments on the number of positive NTC reactions, as compared to no treatment controls, were analysed statistically by Fisher’s exact test. * = P <0.0001. Horizontal bars = median value; percentages = number of positive reactions.

Similar to treatment with EMA or PMA, increasing UV exposure time reduced the persistence of contaminant DNA in reagents, but was also accompanied by a delay in positive reaction Cp and the number of samples detected within the 40 cycle threshold, in a dose-dependent manner. With an exposure time of 2.5 minutes, a reduction of contamination rate from 100% to 5% (n = 42), was accompanied by only 31% detection of 2 *E*. *coli* genome copies (Fig 2).

### A dual UV and EMA decontamination protocol

**Fig 3:**
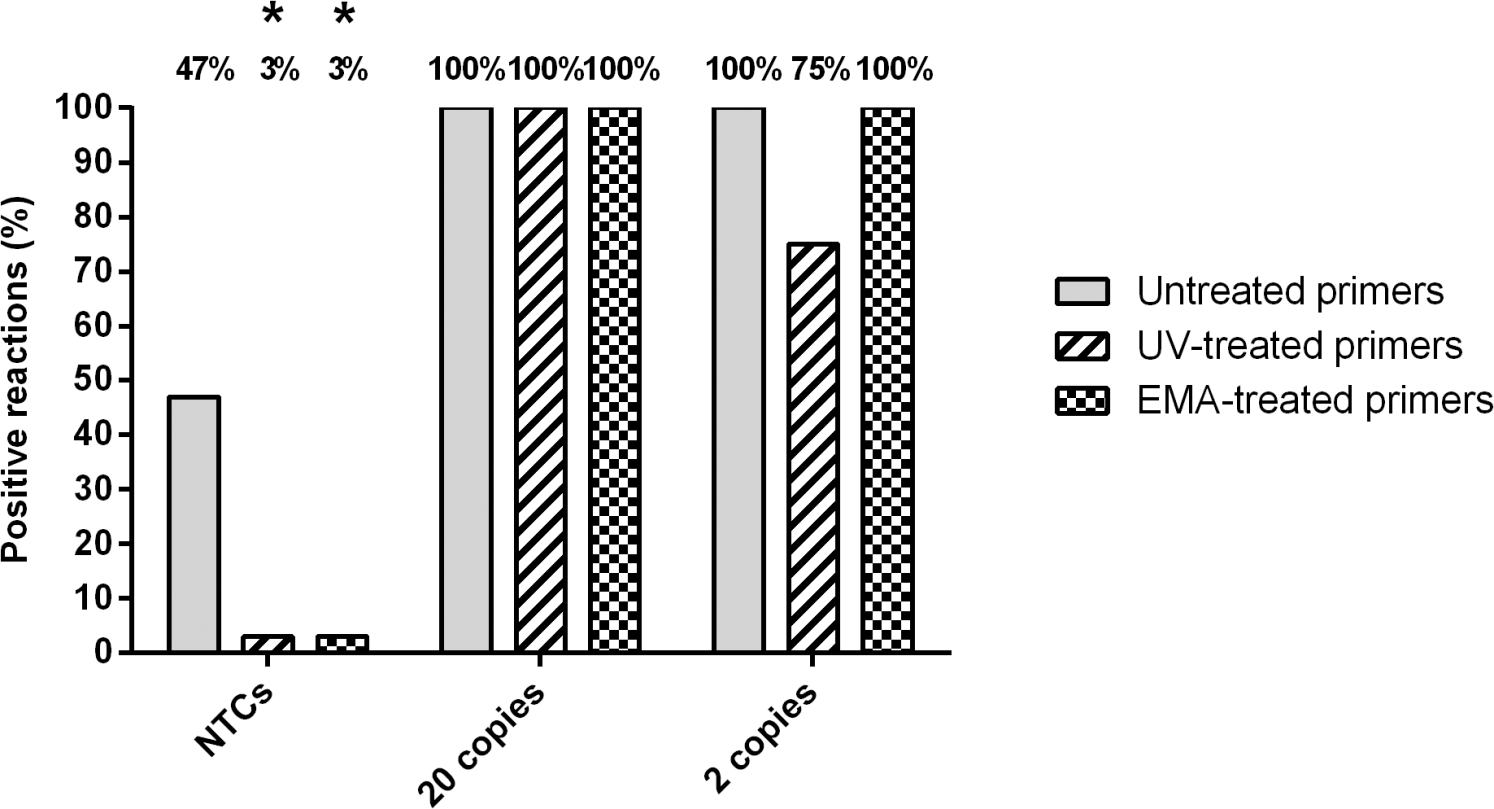
Separate EMA Treatment of Primers. Reactions were repeated with primers added before a two minute UV exposure, compared to post-UV addition of primers treated with or without 1μM EMA. 36 NTC reactions and 12 positive reactions per template concentration were performed for each method. Reactions that did not amplify within the 40 cycle threshold are represented as 40 for visual comparison. Percentages are given for the number of reactions giving a positive result within the 40 cycle threshold. Effects of primer treatments on the number of positive NTC reactions, as compared to non-primer treated controls, were analysed statistically by Fisher’s exact test. * = P <0.0001.

Widely known to induce DNA damage, we hypothesised that UV treatment may impair assay sensitivity through direct effects on the oligonucleotide primers. As such, qPCR experiments were repeated with primers added before and after a two minute UV exposure. Addition of the primers post-treatment saw an unexpected increase in contamination rate from 3% to 47% (n = 36), suggesting that the HPLC-purified primers themselves are a significant source of bacterial DNA contamination. To overcome this issue, primers were treated separately with 1μM EMA, prior to addition to reagents pre-exposed to a UV source for 2 minutes. This dual decontamination method yielded a low contamination rate of 3% (n = 36) and conferred an increase in detection of 2 genome copies from 75% to 100%, compared to UV treatment alone (Fig 3)**.**

To test the analytical sensitivity of the SYBR green qPCR assay and dual decontamination technique on species other than *E*. *coli*, it was employed against a panel of 10 common causative organisms of human bloodstream infections [21]. All species were detectable with 100% frequency at the level of two genome copies per reaction, except for *S*. *epidermidis*, which had a 92% detection rate (n = 12). Template concentrations were diluted further to a theoretical single genome copy per reaction (ranging from 4 to 8 copies of 16S, depending on organism), at which detection rates were more variable, presumably due to the possibility of some reactions containing no template DNA (Table 3). Eight NTC reactions were performed for each species, and contaminant DNA was detected in only 4 of the 80 reactions (5% contamination frequency). Sequencing revealed the DNA to originate from environmental organisms such as *Bradyrhizobiaceae*, *Caulobacter* spp., and *Pelomonas aquatica*, which are likely reagent contaminants, rather than carryover from spiked reactions.

**Table 3:** Detection Rates for Sepsis Panel Organisms.

		Genome copies		
Species		20	2	1
**Gram +ve**	*Enterococcus faecalis*	100%	100%	100%
	*Staphylococcus aureus*	100%	100%	100%
	*Staphylococcus epidermidis*	100%	92%	88%
	*Streptococcus pneumoniae*	100%	100%	88%
**Gram-ve**	*Acinetobacter baumannii*	100%	100%	100%
	*Enterobacter aerogenes*	100%	100%	100%
	*Haemophilus influenzae*	100%	100%	50%
	*Klebsiella pneumoniae*	100%	100%	50%
	*Morganella morganii*	100%	100%	0%
	*Pseudomonas aeruginosa*	100%	100%	38%

The dual UV and EMA decontamination technique was applied to the detection of low amounts of genomic DNA from common sepsis-causing organisms. Numbers show percentages of positive reactions (n = 12).

## Discussion

Commercially available qPCR reagents are recognised to harbour bacterial DNA contamination. While a number of decontamination methods have been described, they often show poor reproducibility or have a negative impact on the analytical sensitivity of broad-range assays designed to detect bacterial DNA [15]. As a result, the clinical utility of PCR-based approaches in infection diagnosis in low-burden conditions, such as in the blood of patients with sepsis, is currently limited. It was therefore the aim of this study to develop a reliable decontamination strategy for a pan-bacterial qPCR assay from commercially available reagents, which would minimise contamination while retaining detection sensitivity of 2 genome copies or less.

Previous studies have demonstrated that light-reactive DNA-intercalating chemicals EMA and PMA can be successfully used as a sole decontamination measure for qPCR reagents, with no positivity in NTC reactions reported, despite use of broad range 16S primers [17,19,20]. There exists some conflicting data though on the importance of amplicon length to the decontamination efficiency, as Schnetzinger et al. (2013) had to increase the product size beyond 1kb for reliable decontamination in contrast to the much shorter 169 bp amplicon employed by Patel et al. (2012). The work presented here supports a positive correlation between amplicon length and decontamination efficiency, presumably due to the increased likelihood of an EMA molecule intercalating within a longer target to render it non-amplifiable by PCR. However, the reductions in PCR efficicency that accompany increased amplicon size, which may also contribute to reduced contaminant detection, introduce a trade-off with the limit of detection of pathogen DNA at low target concentrations, prompting the design of an intermediate sized PCR product (756 bp) in the present study.

There is relatively little information in the literature on total number of negative control reactions performed following EMA or PMA decontamination, with which to assess the extent of persisting amplifiable DNA, which would generate false positive results in a diagnostic setting [17,19,20]. Confidence in the false positive rate of a diagnostic assay is of paramount importance when the assay is formulated, for example, as a rule out test. False positives generated by contaminating bacterial DNA in assay reagents are problematic in clinical diagnostics leading potentially to unnecessary treatment with potent broad spectrum antimicrobials. The large numbers of negative control reactions reported here confirm that neither EMA or PMA gives satisfactory decontamination at concentrations permissive to low level template detection (2 genome copies), when used as the sole decontamination measure. In addition, quite potent PCR inhibition was observed from both EMA and PMA at concentrations ≥0.5 μM which is considerably lower than concentrations reported to be non-inhibitory by other authors [17,20], but may reflect the lower template amounts used here. As primer pairs often respond differently to PCR inhibitors [22], testing of more primer pairs or utilisation of emerging nucleic acid technologies such as LNA [23] could help produce assays that can tolerate higher concentrations of these chemicals. The addition of non-target genomic DNA (e.g. calf-thymus DNA) has also been proposed to ameliorate the inhibitory effects of EMA treatment on a species-specific assay [18], but this could not be evaluated here due to detection of bacterial DNA contamination in the sourced calf-thymus DNA by our universal assay, as experienced by other authors [20].

Although EMA/PMA treatment alone did not effectively eliminate reagent contamination without impairing assay sensitivity, encouraging results were achieved when used to decontaminate HPLC-purified primers separately from other reagents, which could better tolerate parallel decontamination by well-established UV irradiation methods. Removal of contaminant DNA was tested extensively by this combination approach, with DNA persisting in less than 5% (5/116) of NTC reactions performed during the course of this study. We believe that such large numbers of control reactions are imperative when assessing decontamination protocols, and their number should be explicitly stated in manuscripts, which is not always the case [17–20]. The additional time required for the decontamination method presented here could be shortened to approximately 5 minutes through previous batch treatment of primers, but takes only an extra 25 minutes to perform in its entirety. This is much shorter than the elegant, but involved method of broad-range primer extension-PCR (PE-PCR) which increases set up time by more than two hours [24], or the complicated multi-step procedure of Champlot *et al*. requiring reagents to undergo UV and gamma irradiation alongside two enzymatic incubations [16]. Analytical sensitivity is also improved with 100% detection of 2 *E*. *coli* genome copies, compared to only 50% with the PE-PCR method [24], and a 5-fold improved limit of detection over a PMA-only protocol [20]. Indeed, we observed 99% detection across a panel of 10 species commonly causative of sepsis at the level of 2 genome copies per reaction (n = 120). The dye based approach used here also has potential to give limited speciation data if multi-step high resolution melt curve analysis of the product is employed [25].

Several different batches of AmpliTaq Gold 360 Master Mix were used during the course of our investigations, with positive NTC rates fluctuating from 0–5% after the dual UV and EMA treatment (100% positivity without treatment). Such fluctuation suggests the levels of contamination may be near the threshold of what is removable from the reagents by this method, or that small amounts of DNA can be introduced post-treatment, through handling, plasticware or the environment. This background contamination level was deemed acceptable in light of the high analytical sensitivity. However, recent batches of mastermix have shown significantly more persistence of microbial DNA following dual UV-EMA treatment seemingly correlating with the manufacturer’s change of enzyme supplier from early 2014. Sequencing of recent positive NTC products revealed the DNA responsible to originate from members of the *Bradyrhizobiaceae*, *Caulobacter* spp., and *Pelomonas aquatica*, which is consistent with contamination observed in ultrapure water systems [6,26]. Fluctuations in the burden of such environmental organisms can be missed by manufacturing QC processes, which often focus on DNA carryover from recombinant organisms used in enzyme production (e.g. *E*. *coli*) instead. We would like to highlight the need for manufacturers to apply more rigorous QC methods to account for and minimise environmental contamination when producing reagents for broad range applications.

In conclusion, we report a simple, dual decontamination strategy for removal of bacterial DNA from PCR reagents for use with a pan-bacterial primer set. This approach provides a mechanism to improve the signal-to-noise ratio of qPCR-based bacterial detection, allowing detection of low femtogram amounts of pathogen DNA that are often implicated in patients with suspected sepsis, for whom molecular diagnostics with adequate sensitivity are still lacking [27]. However, a fully developed method will also require robust DNA extraction methods from large blood volumes but with small elution volumes, and ultrapure extraction reagents. The described method could also have direct utility in the field of microbial ecology, where PCR contaminants commonly complicate investigations [28,29]. In both settings, the long 16S rRNA gene amplicon designed here would provide considerable speciation power.
